# Phosphorylation of KIAA1429 promotes oxaliplatin resistance through activating the FZD7-Wnt signaling in BRAF^V600E^-mutated colorectal cancer

**DOI:** 10.1186/s13046-025-03449-w

**Published:** 2025-07-03

**Authors:** Taixuan Wan, Minyi He, Zhanzhen Liu, Yihang Zhou, Yebohao Zhou, Wei Xiao, Hao Xie, Shuangling Luo, Haoqi Zheng, Liang Kang, Yunxing Shi, Liang Huang

**Affiliations:** 1 Department of General Surgery (Colorectal Surgery), The Sixth Affiliated Hospital, Sun Yat-sen University, Guangzhou, China; 2 Guangdong Provincial Key Laboratory of Colorectal and Pelvic Floor Diseases, The Sixth Affiliated Hospital, Sun Yat-sen University, Guangzhou, China; 3 Biomedical Innovation Center, The Sixth Affiliated Hospital, Sun Yat-sen University, Guangzhou, China; 4 Department of Gastrointestinal Surgery, The First Affiliated Hospital, Sun Yat-sen University, Guangzhou, China

**Keywords:** CRC, Phosphorylation, Oxaliplatin resistance, m^6^A, BRAF

## Abstract

**Background:**

The current standard therapeutic approach for colorectal cancer (CRC) is surgical operation and oxaliplatin (OXA)-based neoadjuvant chemotherapy. However, the acquisition of oxaliplatin resistance leads to an unfavorable prognosis in CRC. Therefore, there is an urgent need to elucidate the underlying mechanisms of oxaliplatin resistance.

**Methods:**

RNA-sequencing (RNA-seq) analysis of OXA-resistant CRC cell line was used to identify the driver of OXA resistance. Function of KIAA1429 in OXA-resistance was validated by in vivo and in vitro experiments. The underlying mechanism was investigated by Immunoprecipitation-Mass Spectrometry (IP-MS), Co-Immunoprecipitation (Co-IP), Immunofluorescence (IF), RNA immunoprecipitation (RIP) and RNA-seq.

**Results:**

KIAA1429 is significantly upregulated in oxaliplatin-resistant cell lines. However, we found that the expression level of KIAA1429 is not associated with the efficacy of neoadjuvant chemotherapy in colorectal cancer, indicating that the function of KIAA1429 is not solely determined by its expression level. We discovered that KIAA1429 exhibits differential nuclear and cytoplasmic distribution in colorectal cancer samples and that high cytoplasmic expression of KIAA1429 is associated with poor response to chemotherapy. Further investigation revealed that the nuclear-cytoplasmic distribution of KIAA1429 is regulated by BRAF-mediated phosphorylation. In vitro and in vivo experiments indicated that BRAF-mediated phosphorylation of KIAA1429 promotes oxaliplatin resistance by facilitating its aggregation in the cytoplasm. Mechanistically, we found that cytoplasmic KIAA1429 promotes WNT pathway activation by binding and stabilizing FZD7, thereby further enhancing cancer stemness and oxaliplatin resistance.

**Conclusions:**

This study elucidates the unique role of KIAA1429 phosphorylation in regulating its nuclear localization and function, offering novel insights into the mechanisms underlying OXA-resistance in CRC.

**Supplementary Information:**

The online version contains supplementary material available at 10.1186/s13046-025-03449-w.

## Background

Globally, colorectal cancer (CRC) ranks as the third most frequently diagnosed cancer and is the second - leading cause of cancer - related deaths [1]. Oxaliplatin - based chemotherapy is the standard therapeutic approach for CRC patients as recommended by numerous guidelines [2]. Nevertheless, the side effects accompanied with chemotherapy, as well as the inherent and acquired resistance to chemotherapy, can lead to the recurrence of the tumor, its further progression, or the development of distant metastases [3–6]. Therefore, there is an urgent need to clarify the molecular mechanisms underlying chemoresistance and identify effective biomarkers to boost chemosensitivity. This would contribute to mitigate resistance, enhance the prognosis, and prolong the survival of patients with CRC.

The N6-methyladenosine (m^6^A) modification is the most prevalent form of RNA modification [7] m^6^A modification acts as a versatile regulator of gene expression, playing a vital role in RNA metabolism, which influencing RNA stability, splicing, transcription, and degradation in eukaryotic mRNAs. As a reversible modification, it dynamically regulates various physiological and pathological processes. These processes encompass the hematopoietic system, central nervous system, reproductive system, and more [8, 9].Moreover, it is closely related to chemotherapeutic resistance [3, 4, 10]. Vir-like m^6^A methyltransferase-associated (KIAA1429) protein is the largest known component by molecular weight within the m^6^A methyltransferase. KIAA1429 functions as a scaffold that connects the catalytic core components of the methyltransferase complex, including METTL3, METTL14, and WTAP, to RNA substrates. This interaction is crucial for the precise deposition of m^6^A modifications at specific sites [7, 11]. At the same time, KIAA1429 has been confirmed to exert its oncogenic function in multiple cancer types through its m^6^A modification function [12–15]. However, its function in oxaliplatin resistance of CRC has not yet been explored.

BRAF is a kind of serine/threonine kinase, with a mutation rate of approximately 12% in metastatic CRC. Moreover, CRC patients with BRAF mutations often exhibit frequent drug resistance [16]. Its binding substrates has been explored extensively.

In this study, we demonstrate for the first time that KIAA1429 exhibits differential nuclear and cytoplasmic distribution in colorectal cancer samples and that high cytoplasmic expression of KIAA1429 is associated with poor response to chemotherapy. BRAF-mediated phosphorylation of KIAA1429 facilitates its aggregation in the cytoplasm. Furthermore, we found that cytoplasmic KIAA1429 promotes WNT pathway activation by binding and stabilizing FZD7, thereby further enhancing cancer stemness and oxaliplatin resistance. Taken together, our study elucidates the role and key mechanisms of KIAA1429 in oxaliplatin resistance, and provides potential molecular markers for the prediction of neoadjuvant therapy and prognosis in CRC.

## Methods

### Patients and tissues

This study was approved by the Ethics Committee of the sixth affiliated hospital of Sun Yat-Sen University. Tissue samples were retrospectively obtained from CRC patients who received neoadjuvant therapy at the the sixth affiliated hospital of Sun Yat-Sen University, Guangzhou, China, from 2014 to 2021. The study design conformed to the ethical guidelines of the 1975 Declaration of Helsinki.

### Evaluation of treatment efficacy

OS was defined as the interval from date of surgery to date of death or last follow-up. DFS was defined as the interval from date of surgery to date of recurrence or last follow-up. The TRG grade system reflects the extent to which the primary tumor mass in the resection specimen is replaced by fibrosis following neoadjuvant treatment. It is assessed on a scale of 0 to 3, with TRG0 indicating a complete response, where 100% of the tumor is replaced by fibrosis and no viable tumor cells remain. TRG3 signifies a lack of response, meaning there is no fibrosis and the tumor consists entirely of viable cells.

### Cell lines and cell culture

SW480, WiDr and HEK293T were purchased from the Guangzhou jenniobio Biotechnology with STR (short tandem repeat) appraisal certificates. All cell lines were tested negative for mycoplasma contamination. Cells were maintained in Roswell Park Memorial Institute (RPMI) 1640 Medium (Thermo Fisher, USA) supplemented with 10% fetal bovine serum (FBS; Gibco, California, USA) at 37 °C in 5% CO2.

### Cell proliferation, colony formation, and apoptosis assays

For the cell proliferation assay, 1000 cells were seeded into 96-well plates, cell viability was assessed for 5 consecutive days with or without oxaliplatin treatment (1 μm) by the Cell Counting Kit-8 (CCK8) (Dojindo, Japan). For the colony formation assay, 1000 cells were seeded into 6-well plates for about 10 days with or without oxaliplatin treatment (0.5 μm), which were stained with crystal violet and counted at the endpoint. All studies were conducted in triplicates. For the apoptosis assay, cells were treated with oxaliplatin (40 μm) for 48 h. The cells were labeled with Annexin V/APC and 7-AAD (KeyGEN Bio-TECH, China) according to the manufacturer’s instructions.

### RNA-seq and quantitative RT real-time PCR (qPCR)

Total RNA extraction and cDNA synthesis were performed using RNA-Quick Purification Kit (ES Science, Guangzhou, China) and Prime-Script cDNA synthesis kits (Invitrogen, California, USA) according to the manufacturer’s instructions. The cDNA products were used for qPCR analysis using SYBR Green PCR kit (Invitrogen, California, USA). Table S1 includes detailed information about the sequence of the used primers.

### Immunoblotting (IB)

Cells and tissues were lysed with RIPA lysis buffer (MedChemEx-press, HY-K1001). Proteins were extracted and loaded in SDS-PAGE, and transferred onto PVDF membrane (Millipore, Billerica, MA, USA). After blocking with 5% skim milk (Beyotime, China) and sequential incubation with the primary and secondary antibodies (Table S2), the blots were detected using the ECL detection kit (Millipore).

### Immunohistochemical (IHC)

Paraffin-embedded CRC tissues were cut into 5-mm sections. The sections were subsequently incubated with antibodies against human KIAA1429 (Proteintech, Cat No. 25712-1-AP, 1:200), human FZD7 (Proteintech, Cat No. 16974-1-AP, 1:500), human β-catenin (Proteintech, Cat No. 51067-2-AP, 1:2000), anti-Ki67 antibody (Abcam, Ab15580, 1:100) and anti-Cleaved-caspase 3 antibody (Cell Signaling Technology, #9664, 1:200). The IHC score was determined by evaluating two parameters under light microscopy. Staining intensity, reflecting the depth of color, was subjectively graded on a scale of 0 (none) to 3 (strong), with intermediate scores of 1 (weak) and 2 (moderate). The percentage of positive cells, indicating the extent of protein expression, was categorized and scored from 1 to 4, corresponding to 0–25%, 26–50%, 51–75%, and 76–100% respectively. The final IHC score was obtained by multiplying these two individual scores. All evaluations were performed by two independent observers who were unaware of the clinical outcomes. The definition of KIAA1429 nuclear localization is: KIAA1429 protein shows positive staining (≥ 2) in > 50% of tumor cells, with weak or absent staining in the cytoplasm. Otherwise, cytosolic localization is defined.

### RNA-immunoprecipitation-quantitative PCR (RIP-qPCR)

RNA immunoprecipitation (RIP) assays were conducted using both the Magna RIP™ RNA-Binding Protein Immunoprecipitation Kit (Millipore, Bedford, MA), following the manufacturer’s instructions with slight modifications to optimize for antibody specificity and RNA recovery. Briefly, cells were lysed in RIP lysis buffer containing RNase inhibitors, protease inhibitors, and DTT, and incubated on ice for 10–15 min. The lysates were then centrifuged at 12,000 × g for 10 min at 4 °C to remove cell debris. The clarified supernatant was collected and transferred into fresh tubes. For immunoprecipitation, 40 µL of Protein G magnetic beads were pre-washed twice with ice-cold IP buffer and then added to the cell lysates along with 5 µg of specific antibody. The antibodies used in this study included human anti-KIAA1429 (1:50, Proteintech, ab181861) and normal rabbit IgG (Millipore) as a negative control. The samples were incubated overnight at 4 °C on a rotator to allow the formation of RNA-protein-antibody complexes. After incubation, magnetic separation was used to collect the beads, and the supernatant was discarded. The beads were then washed four times with 1× RIP wash buffer, each time resuspending and rotating for 10 min at 4 °C to minimize nonspecific interactions. To digest the protein component and release the co-precipitated RNA, the beads were treated with proteinase K at 55 °C for 30 min. RNA was then extracted using TRIzol™ Reagent (Invitrogen™, Cat#15596018) following the phenol-chloroform extraction method. The RNA pellet was washed with 75% ethanol, air-dried briefly, and resuspended in RNase-free water. The purified RNA was subsequently subjected to quantitative real-time PCR (qRT-PCR) to assess the enrichment of specific transcripts associated with the immunoprecipitated RNA-binding proteins. All RIP-qPCR results were normalized to the input control (10% of total RNA from the lysate) to account for sample-to-sample variability.

### Nuclear-cytoplasmic fractionation

Nuclear and cytoplasmic proteins were extracted using the Nuclear and Cytoplasmic Protein Extraction Kit (KGB5302-50/100, Kegene Biotech) according to the manufacturer’s instructions. For cultured cells, 5 × 10⁶ to 1 × 10⁷ cells were collected by centrifugation at 800 × g for 3 min at 4 °C and washed twice with cold PBS. The pellet was resuspended in 500 µL of ice-cold Buffer A and Buffer B (450 µL + 50 µL, supplemented with PMSF and protease inhibitors), and incubated on ice for 30 min. Lysates were centrifuged at 3,000 rpm for 10 min at 4 °C, and the supernatant was collected as the cytoplasmic protein fraction.The nuclear pellet was resuspended in 100 µL of Buffer C (supplemented with PMSF and protease inhibitors), vortexed for 15 s, and incubated on ice for 30–60 min with vortexing every 10 min. Samples were centrifuged at 14,000 × g for 30 min at 4 °C, and the supernatant was collected as the nuclear protein fraction. For tumor tissue samples, approximately 50 mg of fresh or frozen tumor tissue was minced into small pieces and homogenized in 900 µL Buffer A and 100 µL Buffer B (pre-supplemented with PMSF and protease inhibitors) on ice. After incubation on ice for 30 min, samples were centrifuged at 3,000 rpm for 10 min at 4 °C. The supernatant was collected as the cytoplasmic fraction, and the remaining pellet was further lysed with 200 µL Buffer C as described above to obtain the nuclear protein fraction. Protein concentrations were measured using the BCA assay. All protein samples were aliquoted and stored at − 80 °C to avoid repeated freeze-thaw cycles.

### Small interfering RNAs (siRNA)

Small interfering RNAs (siRNA) for BRAF, FZD7 were purchased from Genepharma (A10001). Transfection of small interfering RNA was performed with Lipofectamine-RNAiMAX (Invitrogen, 13778100). After 6–8 h, the supernatant was replaced with fresh medium and the efficiency was identified by qRT-PCR and western blotting 72 h after transfections. Targeting sequences were listed in Supplementary Table S3.

### Immunofluorescence (IF)

For IF analysis of cultured cells, CRC/293T cells were grown on chamber slides precoated with poly (L-lysine). Cells were fixed with cold paraformaldehyde. Cultured cells were permeabilized with PBS containing 0.1% Triton X-100, and blocked with AquaBlock (East Coast Bio, PP82). Cells were probed with the following primary antibodies as following: anti-KIAA1429 (Proteintech, 25712-1-AP, 1:200), anti-BRAF (Proteintech, 15282-1-AP, 1:200), anti-CoraLite^®^ Plus 488-conjugatedKIAA1429(Proteintech, CL488-68235,1:200), anti-β-catenin (Proteintech, 51067-2-AP, 1:500) anti-Phosphoserine/Threonine Pure 22a (BD Biosciences, 612549, 1:200) (Supplementary Table S2). After washing the cells with PBS-T three times, the cells were incubated with 594 (or 488) labeled secondary antibodies (1:200) and DAPI-containing mounting solution VECTASHIELD (Vector Laboratories, Vector H-1000). The slices were visualized by using a Nikon inverted microscope Eclipse Ti-U equipped with a digital camera, or a Nikon A1 laser scanning confocal microscope at the Center for Advanced Microscopy/Nikon Imaging Center (CAM).

### Immunoprecipitation (IP), mass spectrometry analysis of KIAA1429 interacting proteins

Cells and tissues were lysed using IP lysis buffer (Thermo Fisher Scientific, #87787) supplemented with proteinase inhibitor (Sigma-Aldrich, #04693132001) for 10 min, and cleared by centrifugation at 12,000 x g at 4 °C for 20 min. Cell lysate (2 mg) was subjected to IP with the indicated antibodies overnight at 4 °C. Then the lysate was incubated with protein A/G agarose beads (Thermo Fisher Scientific, #88803) for 1 h at room temperature. The beads were washed 3 times with IP lyse buffer. Then the proteins were eluted with loading buffer and detected by western blotting. For Mass spectrum analysis of KIAA1429 interaction, the peptides were extracted and evaporated for liquid chromatography-mass spectrometry (LC-MS) analysis at the FITGENE company (Guangzhou, China).

### RNA stability assay

CRC cell lines were plated in 6-well plates and exposed to actinomycin D (5 µg/mL, Sigma) for 0, 2, 4, 6 and  8h. RNA was extracted at the indicated times and analyzed by qPCR.

### Bioinformatics analysis

Data from RNA-seq were analyzed by R (V3.3, http://www.bioconductor.org) with the edge R package. Fold-change (FC) of gene expression was calculated with threshold criteria of FC ≥ 2 and *P* value < 0.05. Enrichment analysis was performed to investigate the processes of the candidate genes or metabolites, by applying online tools of the DAVID (https://david.ncifcrf.gov/). Public data originated from GEPIA (http://gepia.cancer-pku.cn/) or KM plotter databases ( https://kmplot.com/analysis/).

### Animal experiments

All animal experiments were approved by the Institutional Animal Care and Use Committee of the Sixth Affiliated Hospital, Sun Yat-Sen University. BALB/C nude mice were kept in an animal room with a 12-hour light-dark cycle at a temperature of 20–22 °C with 40–70% humidity. For the subcutaneous tumor models, 5 × 10^6^ SW480 cells were injected into 4-week-old male BALB/C nude mice, and the tumor tissues were taken out 1 month later for future experiments. Drug was adopted when the tumors reached about 50 mm^3^ in size, at which point mice were randomized for treatment with PBS (intraperitoneally), oxaliplatin (5 mg/kg/every 3 days, intraperitoneally).

### Statistics and reproducibility

Statistical analysis was performed using GraphPad Prism version 8.0 for Windows. For comparing two groups, the two-tailed Student t-test was used unless otherwise stated. One-way ANOVA analyzed for multiple comparisons. Experiments were performed a minimum of three times. *P* < 0.05 was considered statistically significant. All grouped data are presented as mean ± SD unless otherwise stated. *: *p* < 0.05; **: *p* < 0.01.

## Results

### The differential nuclear and cytoplasmic distribution of KIAA1429 is correlated with chemotherapeutic response and prognosis in CRC patients

**Fig. 1:**
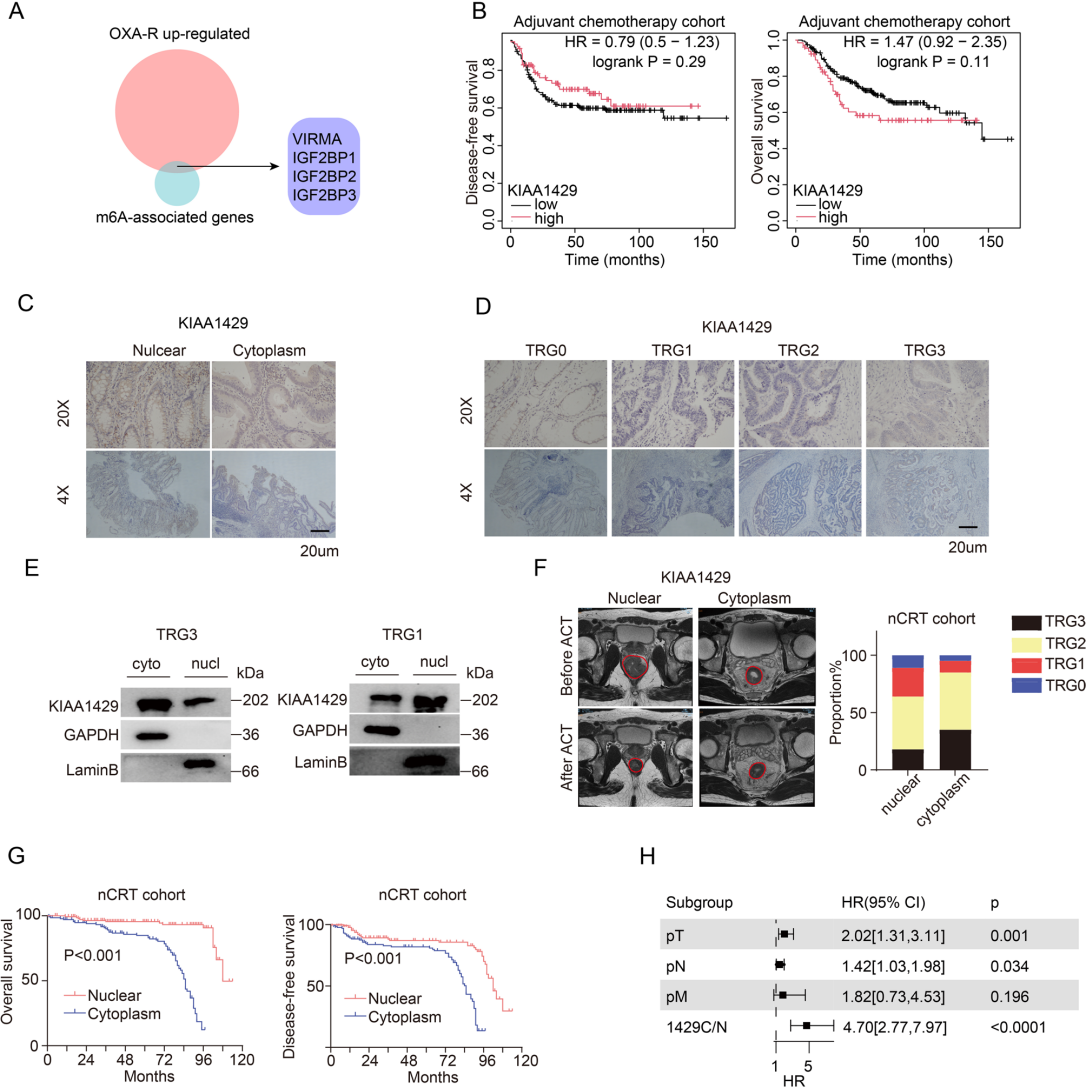
The differential nuclear and cytoplasmic distribution of KIAA1429 is correlated with chemotherapeutic response and prognosis in CRC patients **(A)** Venn diagram shows the intersection of genes upregulated in oxaliplatin resistance and m^6^A-related genes. **(B)** The K-M plotter database illustrates the prognosis of postoperative chemotherapy in patients with high and low levels of KIAA1429. (DFS: Group High *n* = 83 Group Low *n* = 195; OS: Group High = 65 Group Low = 191). **(C)** Representative images of the cytoplasmic and nuclear distribution of KIAA1429. Scale bar, 20 μm. **(D)** Representative immunohistochemical images of patients who received neoadjuvant chemotherapy, categorized by different Tumor Regression Grade (TRG) classifications. Scale bar, 20 μm. **(E)** The representative nuclear-cytoplasmic fractionation Western Blot experiments from patient-derived samples. (*n* = 3 biologically independent samples). **(F)** The representative MRI images of patients dependent on KIAA1429 cytoplasmic and nuclear distribution and bar charts of cohort TRG distribution. **(G)** Overall survival and Disease-free survival of SYSU6 Cohort grouped by KIAA1429 cytoplasmic and nuclear distribution. (Cytoplasm: *n* = 126; Nuclear: *n* = 155) **(H)** Forest diagram showing the hazard ratio (HR) and 95% confidence interval (CI) based on multivariate regression analysis of overall survival in patients with CRC. Data are represented as mean ± SD. OXA, oxaliplatin; TRG, tumor regression grade; nCRT, neoadjuvant therapy

To elucidate the molecular mechanisms underlying oxaliplatin resistance, we identified genes related to m^6^A modification that were upregulated in oxaliplatin-resistant cell lines. These genes included the m^6^A “reader” proteins IGF2BP1, IGF2BP2, IGF2BP3, and VIRMA (KIAA1429). Given that our team had previously investigated the IGF2BP family member [3], we focused our attention on KIAA1429 (Fig. 1A). Subsequently, analysis based on the K-M Plotter database revealed that the expression level of KIAA1429 was not a key prognostic factor for adjuvant chemotherapy outcomes (Fig. 1B). However, building on prior finding that the unique distribution of KIAA1429 in the cytoplasm and nucleus of breast cancer cells influences tumor prognosis [17], we hypothesized that the subcellular localization of KIAA1429 might similarly impact chemotherapeutic efficacy in CRC(Fig. 1C).To test this hypothesis, we retrospectively analyzed pre-treatment specimens from locally advanced CRC patients who underwent neoadjuvant therapy between 2014 and 2021 using immunohistochemical methods. Intriguingly, we found that the nuclear distribution of KIAA1429 was significantly associated with tumor regression grade (TRG) following neoadjuvant therapy in CRC patients (Fig. 1D, E, F; Table S4). Specifically, an increased nuclear localization of KIAA1429 correlated with more pronounced tumor regression, suggesting a potential role in therapeutic response. Moreover, in the neoadjuvant therapy cohort, patients with nuclear localization of KIAA1429 exhibited better prognosis compared to those with cytoplasmic localization (Fig. 1G). Multivariate Cox regression analysis further confirmed that the subcellular localization of KIAA1429, particularly its nuclear versus cytoplasmic distribution, significantly impacted patient outcomes (Fig. 1H). In summary, our findings suggest that differential nuclear and cytoplasmic distribution of KIAA1429 can serve as a novel biomarker to predict both chemotherapeutic efficacy and prognosis in CRC patients.

### Phosphorylation of KIAA1429 at S1579 regulates its cytoplasmic localization

**Fig. 2:**
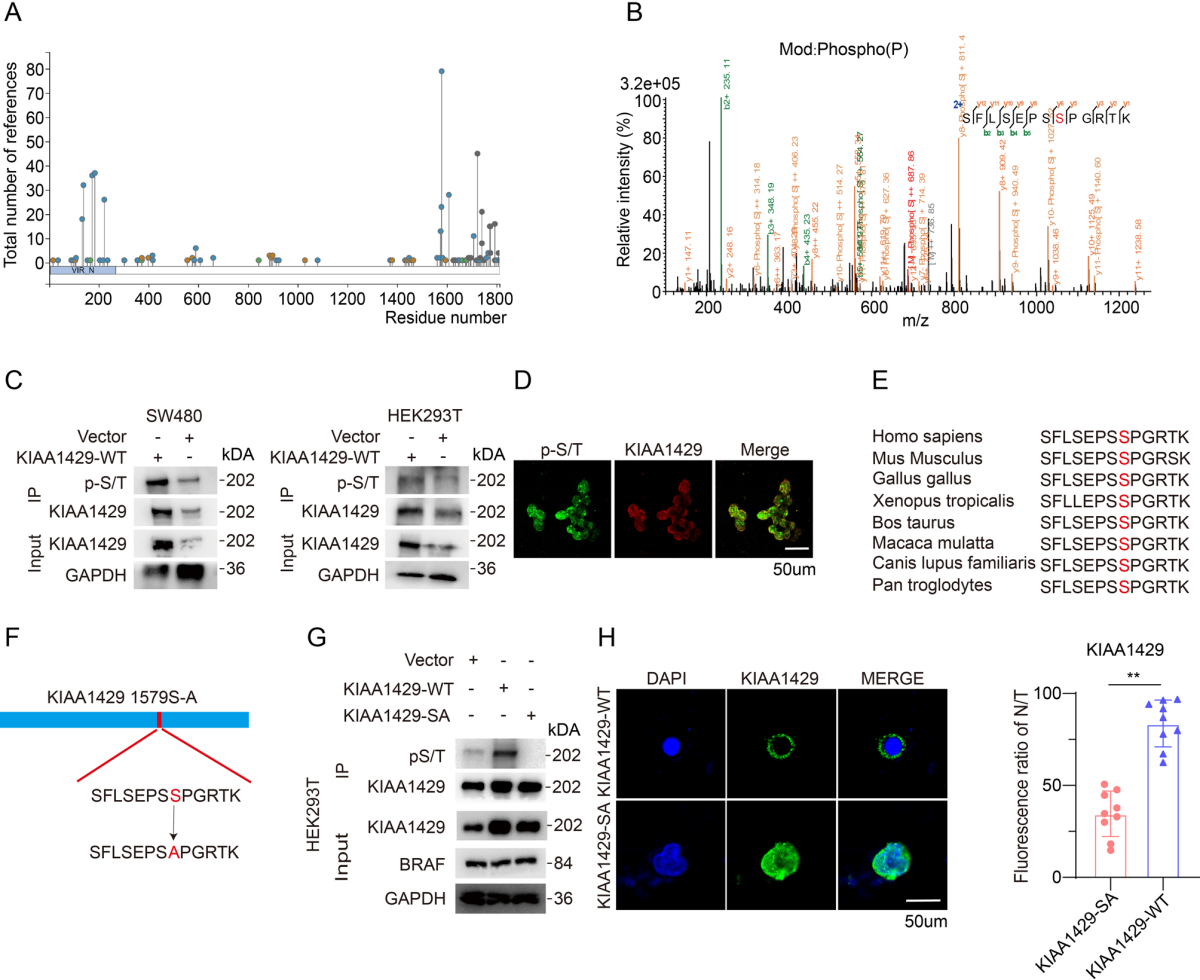
Phosphorylation of KIAA1429 at S1579 regulates its cytoplasmic localization **(A)** Prediction of post-translational modifications on the KIAA1429 by phosphositeplus database. **(B)** Phosphorylation modification of KIAA1429 identified by liquid chromatography/tandem mass spectrometry (LC-MS/MS). **(C)** Western blot analysis of immunoprecipitated KIAA1429 was performed using a pan-serine/threonine (pS/T) antibody to assess the impact of KIAA1429 overexpression on its phosphorylation levels in SW480 and HEK293T cells. (*n* = 3 biologically independent samples) **(D)** Immunofluorescence staining showed the co-localization of p-S/T (green) and KIAA1429 (red) in SW480. Scale bar, 50 μm. **(E)** The sequences surrounding S1579 of KIAA1429 are evolutionarily conserved across multiple species. **(F)** Schematic diagram of the mutation of serine at position 1579 of KIAA1429 to alanine. **(G)** WB analysis of immunoprecipitated KIAA1429 showed that S1579A mutation dramatically reduced pS/T in HEK293 cells after overexpressing KIAA1429-WT or KIAA1429-SA (*n* = 3 biologically independent samples). **(H)** Immunofluorescence staining showed KIAA1429-SA (green) mutant with more obviously nuclear distribution. Scale bar, 50 μm. Data were analyzed by two-sided Student’s t test in H

Given the pivotal role of KIAA1429 in predicting the efficacy of neoadjuvant therapy for colorectal cancer, we initiated a preliminary investigation into its expression across various colorectal cancer cell lines. Western blot analysis revealed that KIAA1429 expression levels were relatively consistent across different colorectal cancer cell lines, with no significant differences observed (Fig. S1). Drawing on our extensive experience in protein post-translational modifications (PTMs) [3, 18], we focused on potential PTMs of KIAA1429. The PhosphositePlus database indicated a significant phosphorylation modification on KIAA1429 [19] (Fig. 2A). To further explore this, we conducted immunoprecipitation followed by mass spectrometry (IP-MS), which identified a notable phosphorylation site at serine 1579 of the KIAA1429 molecule (Fig. 2B). To validate this phosphorylation modification, we transfected SW480 and HEK293T cell lines with wild-type KIAA1429 plasmid or an empty vector. Overexpression of the wild-type KIAA1429 plasmid led to a significant upregulation of pan-serine/threonine phosphorylation (pS/T) on KIAA1429 (Fig. 2C). Immunofluorescence experiments further revealed substantial colocalization of pS/T and KIAA1429 in the cytoplasm (Fig. 2D). Additionally, sequence analysis showed that serine 1579 of KIAA1429 is highly conserved across multiple species during evolution (Fig. 2E). Based on these findings, we constructed a mutant plasmid in which serine 1579 of KIAA1429 was replaced with alanine (1579S) (Fig. 2F). Subsequent overexpression of wild-type KIAA1429 (KIAA1429-WT) and the mutant plasmid (KIAA1429-SA) in HEK293T cells demonstrated a significant downregulation of pS/T modification at the 1579S site (Fig. 2G). Intriguingly, immunofluorescence experiments in SW480 cells transfected with KIAA1429-WT and KIAA1429-SA revealed marked differences in the cytoplasmic and nuclear localization of the KIAA1429 molecule (Fig. 2H). Collectively, these results indicate that serine 1579 of KIAA1429 undergoes phosphorylation, and this modification is a critical regulator influencing its nuclear and cytoplasmic distribution.

### Cytoplasmic accumulation of KIAA1429 mediated by S1579 phosphorylation facilitates oxaliplatin resistance

**Fig. 3:**
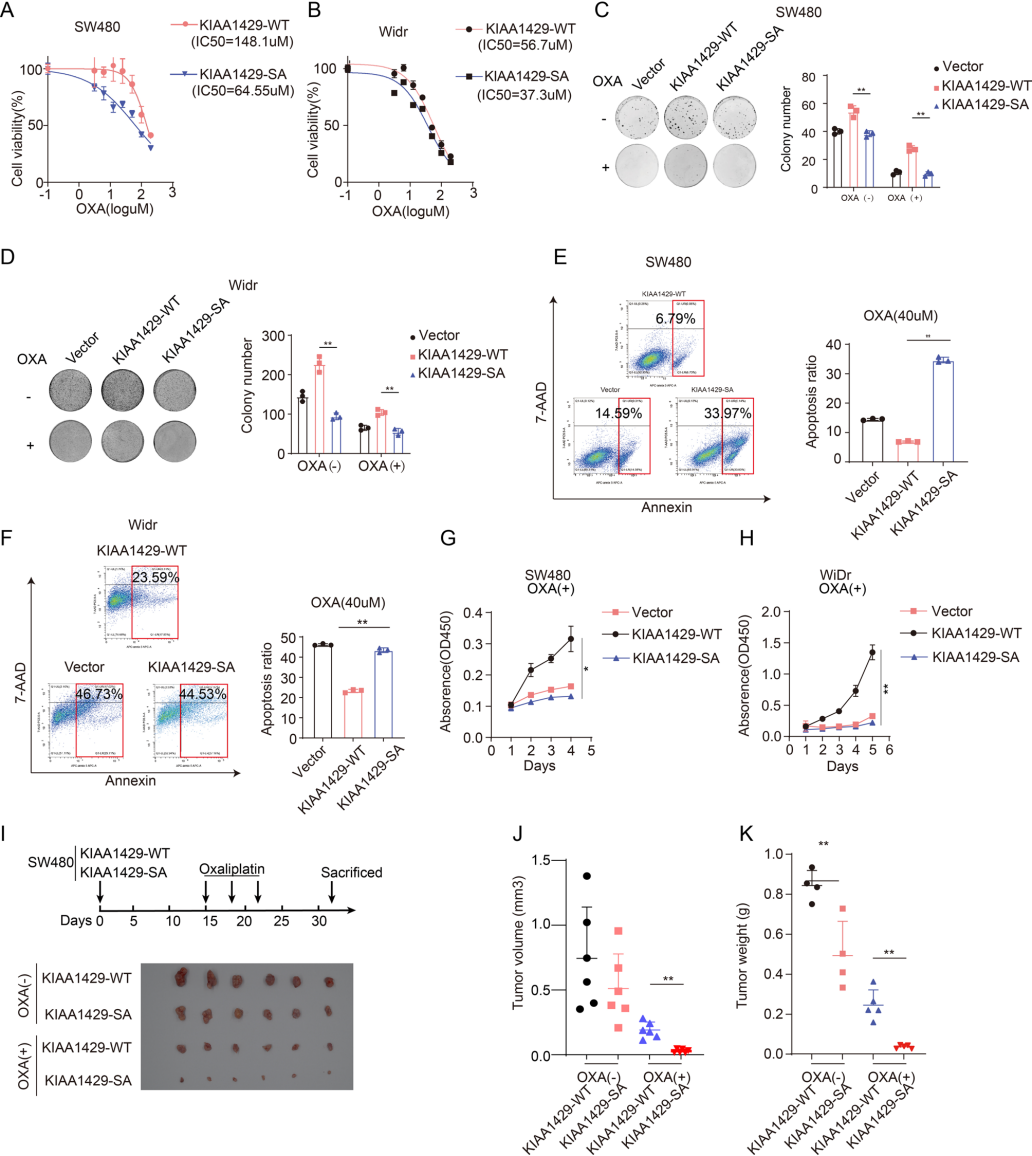
Cytoplasmic accumulation of KIAA1429 mediated by S1579 phosphorylation facilitates oxaliplatin resistance **A-B**. The IC50 of OXA in the KIAA1429-WT/KIAA1429-SA on SW480/ WiDr cells. **C-F.** The effects of KIAA1429-WT and KIAA1429-SA on cell growth (0.5 μm OXA) as shown by colony formation, and on apoptosis (40 μm OXA) by flow cytometry analysis. **G-H.** CCK8 assay to measure the effects of KIAA1429-WT and KIAA1429-SA on cell proliferation (1 μm OXA). **I.** The effect of KIAA1429-WT on the tumor growth of subcutaneously implanted SW480 cells compared to KIAA1429-SA treated with OXA (5 mg/kg) or vehicle control in nude mice (*n* = 6). The measurement of tumor volumes (**J**) and tumor weights (**K**) of KIAA1429-WT cells and KIAA1429-SA cells treated with OXA (5 mg/kg) or vehicle control (*n* = 6). Data were analyzed by one-way ANOVA adjusted for multiple comparisons for C, D,E, F,G, J,K

To this end, we knocked out KIAA1429 in SW480 and WiDr cell lines and subsequently overexpressed either wild-type KIAA1429 (KIAA1429-WT) or the phosphorylation-deficient mutant (KIAA1429-SA) in these cells (Fig. S2). Our results revealed that the IC50 value for oxaliplatin was significantly higher in the KIAA1429-WT group compared to the KIAA1429-SA group, indicating enhanced drug resistance in the former (Fig. 3A, B). Consistent with this observation, colony formation assays demonstrated that KIAA1429-WT overexpressing cells exhibited greater proliferative capacity than their KIAA1429-SA counterparts, irrespective of oxaliplatin treatment (Fig. 3C, D). Furthermore, overexpression of KIAA1429-SA significantly increased oxaliplatin-induced apoptosis in both SW480 and WiDr cell lines, as evidenced by apoptosis assays (Fig. 3E, F). This finding was corroborated by CCK8 assay, which confirmed that KIAA1429-WT overexpressing cells had a significantly stronger proliferative ability than those overexpressing KIAA1429-SA (Fig. 3G, H). To further elucidate the role of KIAA1429 in promoting oxaliplatin resistance in colorectal cancer in vivo, we established subcutaneous tumor models in mice using SW480 cells overexpressing either KIAA1429-WT or KIAA1429-SA (Fig. 3I). Tumor growth was monitored by measuring the time, weight, and volume of subcutaneous tumors. Notably, both the weight and volume of tumors in the KIAA1429-WT group were significantly greater than those in the KIAA1429-SA group (Fig. 3J, K). The immunohistochemical staining of Ki67 and cleaved caspase-3 in mouse tumor sections also confirmed that KIAA1429-WT exhibited faster proliferation than KIAA1429-SA (Fig. S3). In conclusion, these findings highlight that cytoplasmic accumulation of KIAA1429 drive the development of oxaliplatin resistance in colorectal cancer.

### KIAA1429 is phosphorylated at S1579 by BRAF

**Fig. 4:**
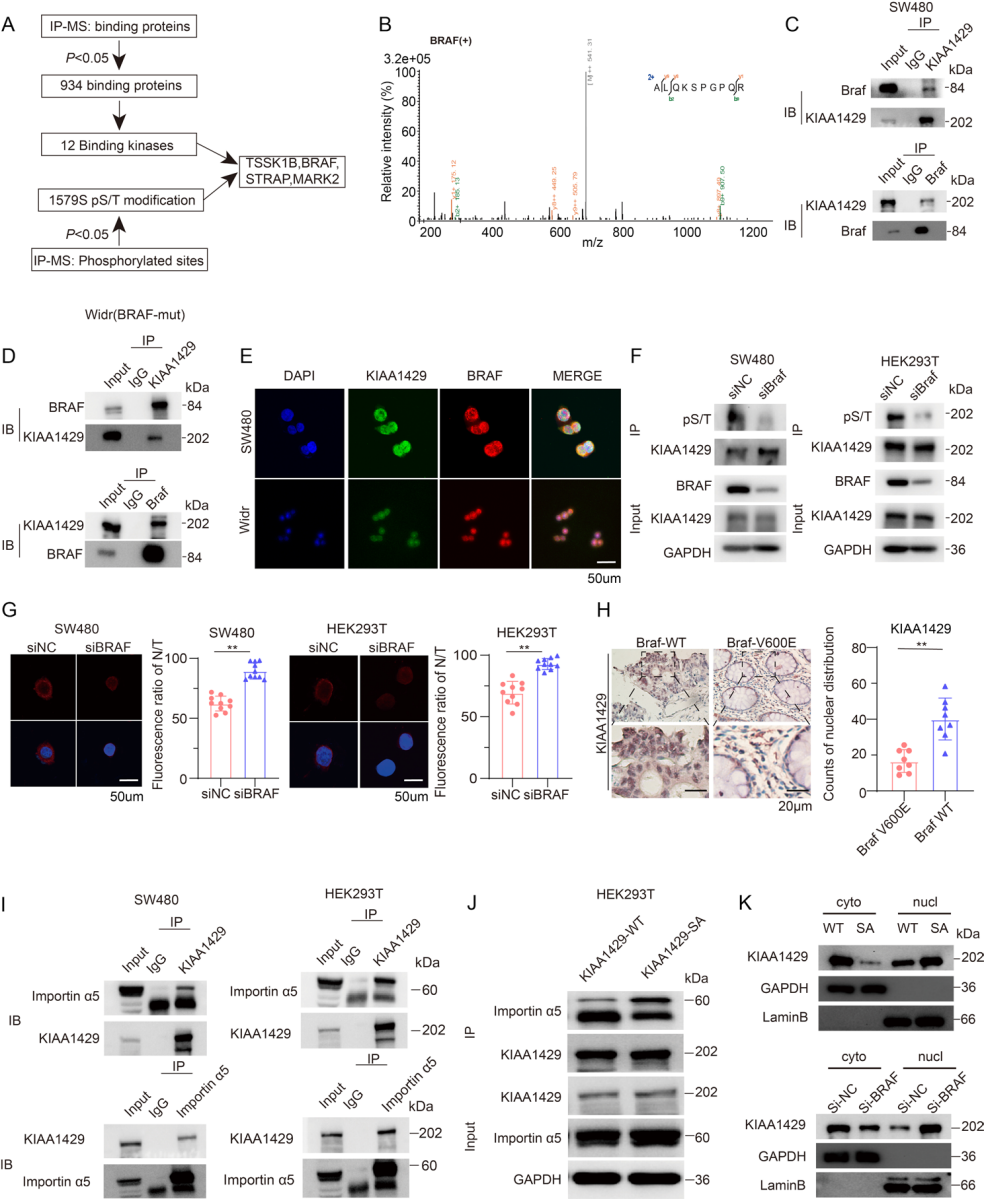
KIAA1429 is phosphorylated at S1579 by BRAF **(A)** Flowchart for identifying BRAF as an upstream kinase for KIAA1429. **(B)** Fragmentation spectrum of the BRAF peptide identified by liquid chromatography/tandem mass spectrometry (LC-MS/MS). **C-D.** WB analysis showed that endogenous BRAF and KIAA1429 interact with each other in SW480 and BRAF-mutant WiDr cells using reciprocal co-immunoprecipitation. **E.** Immunofluorescence staining showed the co-localization of BRAF (red) and KIAA1429 (green) in SW480 Cells and WiDr cells (*n* = 3 biologically independent experiments). Scale bar, 50 mm. **F.** WB analysis of immunoprecipitated KIAA1429 to determine the effect of siBRAF on phosphorylation of KIAA1429 in SW480/HEK293T cells using pS/T antibody (*n* = 3 biologically independent samples). **G.** Immunofluorescence staining showed the KIAA1429 with more obviously cytoplasmic distribution after siBRAF compared to siNC (*n* = 3 biologically independent experiments). Scale bar,50 μm. **H.** Immunohistochemistry demonstrate that KIAA1429 exhibits greater cytoplasmic distribution in patients with BRAF V600E mutations than BRAF-WT. Scale bar,20 μm. **I.** WB analysis showed that endogenous importin α5 and KIAA1429 interact with each other in SW480 and HEK293T cells using reciprocal co-immunoprecipitation. **J.** WB analysis showed that KIAA1429SA has a stronger binding affinity with importin α5 compared to KIAA1429WT (*n* = 3 biologically independent samples). **K**. The nuclear-cytoplasmic fractionation Western Blot experiments demonstrated that KIAA1429 exhibits increased nuclear localization in the KIAA1429SA group and after BRAF knockdown. Data were analyzed by two-sided Student’s t test in G, H

To further explore the potential phosphorylation mechanisms on the KIAA1429 molecule, we performed IP-MS to identify potential binding proteins with KIAA1429. After filtering by *p* < 0.05, 934 proteins were identified. Among the 934 binding proteins, there were 12 kinases. Considering the significant serine phosphorylation modification at position 1579 on KIAA1429, we then screened out four potential serine kinases, including BRAF, a well-known mutated kinase in colorectal cancer. Therefore, we wanted to further explore the effect of BRAF on the phosphorylation of KIAA1429 (Fig. 4A, B). The CO-IP experiment in SW480 and WiDr cell lines (a BRAF-mutated CRC cell line) confirmed the interaction between BRAF and KIAA1429 (Fig. 4C, D). IF co-localization experiments also confirmed this (Fig. 4E). Next, we knocked down BRAF in HEK293T and SW480 cell lines, and the IP experiment also confirmed that the phosphorylation modification on KIAA1429 showed a downward trend after BRAF knockdown (Fig. 4F). Consequently, we were intrigued to explore if the nuclear localization of KIAA1429 is modulated by BRAF. Immunofluorescence experiments were conducted to verify the distinct distribution patterns of KIAA1429 in BRAF wild-type (WT) and BRAF siRNA (si) groups and the results showed that the nuclear localization of KIAA1429 exhibited a marked increase after BRAF knockdown (Fig. 4G). we also selected some postoperative pathological specimens from patients for immunohistochemical (IHC) staining. The IHC results also showed that in patients with BRAF-V600E mutation, KIAA1429 with more obvious cytoplasmic distribution compared to patients with BRAF WT (Fig. 4H). Subsequently, we further explored the mechanism of KIAA1429 nuclear localization. In the IP-MS results of SW480, we identify the nuclear transport protein importin α5 (Fig S4A). Co-IP experiments were conducted in HEK293T and SW480 cell lines, and we found that KIAA1429 interacted with importin α5 (Fig. 4I). Similarly, in the 293T and SW480 cell line, overexpression of KIAA1429 wild-type and mutant plasmids was performed, and the IP experiment showed that the binding of KIAA1429-SA to importin α5 significantly increased, indicating that KIAA1429-SA was transported more into the cell nucleus (Fig. 4J; S4B). The nuclear-cytoplasmic fractionation Western Blot experiment showed that the KIAA1429-WT group exhibited more cytoplasmic distribution compared to the KIAA1429-SA group. Similarly, after BRAF knockdown, KIAA1429 was also significantly distributed in the cytoplasm (Fig. 4K). In summary, these results suggest that KIAA1429 is phosphorylated by BRAF, and BRAF-mediated phosphorylation has a significant impact on the nuclear localization of KIAA1429.

### Cytoplasmic accumulation of KIAA1429 activates WNT signaling by stabilizing mRNA of FZD7

**Fig. 5:**
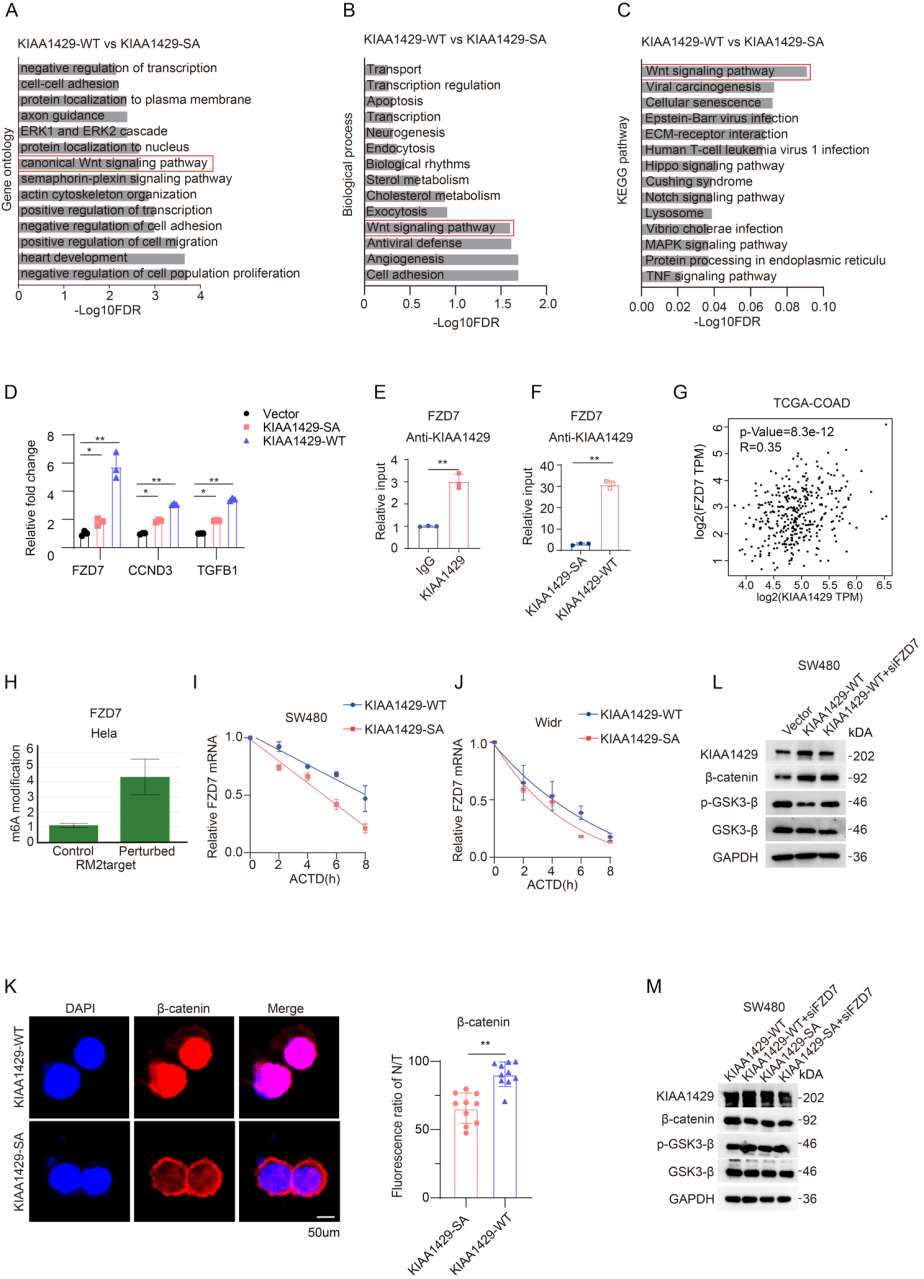
Cytoplasmic accumulation of KIAA1429 activates WNT signaling by stabilizing mRNA of FZD7 **A-C**. The top 10 gene ontology (GO)/Biological process (BP)/Kyoto Encyclopedia of Genes and Genomes (KEGG) terms for DEGs from RNA-seq analysis of KIAA1429-WT and KIAA1429-SA SW480 cells. **D.** qPCR to identify possible target gene for KIAA1429 involved in WNT signaling pathway. **E.** RIP assays demonstrated that the KIAA1429 antibody has a stronger binding affinity with FZD7 than IgG. **F.** RIP assays reveals that the KIAA1429WT has a stronger binding affinity with FZD7 than KIAA1429SA. **G.** GEPIA database showed that KIAA1429 has strong correlation with FZD7. **H.**RM2target database perturbed that KIAA1429 mediates more m^6^A modifications in the Hela cell line. **I-J.** RNA decay assay showed KIAA1429WT maintain the mRNA stability better than KIAA1429SA. **K.** Immunofluorescence experiments demonstrated the β-catenin with more nuclear distribution in KIAA1429WT than KIAA1429SA. Scale bar, 50 μm. **L-M.** Western Blot analysis provided compelling evidence that the KIAA1429–FZD7 axis plays a critical role in mediating the activation of the WNT signaling pathway. Data were analyzed by two-sided Student’s t test in E, F and J

To further explore the mechanism by which KIAA1429 promotes oxaliplatin resistance in CRC, the KIAA1429-WT plasmid and the KIAA1429-SA plasmid were overexpressed respectively into SW480 cells and performed RNA-seq analysis. Gene enrichment analysis was conducted by utilizing the DAVID database. In the KEGG, GO, and BP analyses, we found that the WNT Signaling pathway was present among the top ten upregulated pathways (Fig. 5A-C). The WNT pathway is a classic pathway that regulates cell proliferation and stemness and also closely related to oxaliplatin resistance. Next, we performed QPCR analysis on several upstream genes of the WNT pathway. Among them, FZD7 (frizzled receptor 7) was upregulated by more than five times in the WT group (Fig. 5D). FZD7 is a receptor of the canonical WNT pathway and plays a key role in the activation of the WNT pathway [20]. The RNA immunoprecipitation experiment (RIP) also confirmed this (Fig. 5E). Moreover, the RIP experiments overexpressing WT and SA showed that the WT bound to FZD7 RNA about 30 times more than SA (Fig. 5F). This indicates that only KIAA1429-WT can significantly regulate the function of the WNT pathway. In the TCGA database, KIAA1429 and FZD7 were significantly positively correlated (Fig. 5G). The RM2target database also predicts that KIAA1429 can enhance the m^6^A modification on FZD7 (Fig. 5H). In the SW480 cell line, RNA decay assays showed that overexpression of KIAA1429 resulted in greater RNA stability for the KIAA1429-WT variant compared to KIAA1429-SA; this trend was similarly observed in the WiDr cell line (Fig. 5I-J). Moreover, the immunofluorescence experiment revealed that cells overexpressing KIAA1429-WT exhibited a significantly higher amount of β-catenin translocating into the nucleus compared to those overexpressing KIAA1429-SA (Fig. 5K). The Western Blot experiments also confirmed the relationship between the KIAA1429-FZD7 axis and activation of the WNT pathway. In the KIAA1429-WT groups, the phosphorylated form of GSK-3β was significantly reduced [21, 22](Fig. 5L-M). The stemness assay further demonstrated that KIAA1429-WT markedly enhances the stemness of colorectal cancer cells to a greater extent than KIAA1429-SA (Figure S5). These results imply that FZD7 is a target gene of KIAA1429, cytoplasmic accumulation of KIAA1429 promotes oxaliplatin resistance in colorectal cancer through activating the WNT pathway.

### The effects of cytoplasmic KIAA1429 on OXA resistance is dependent on FZD7

**Fig. 6:**
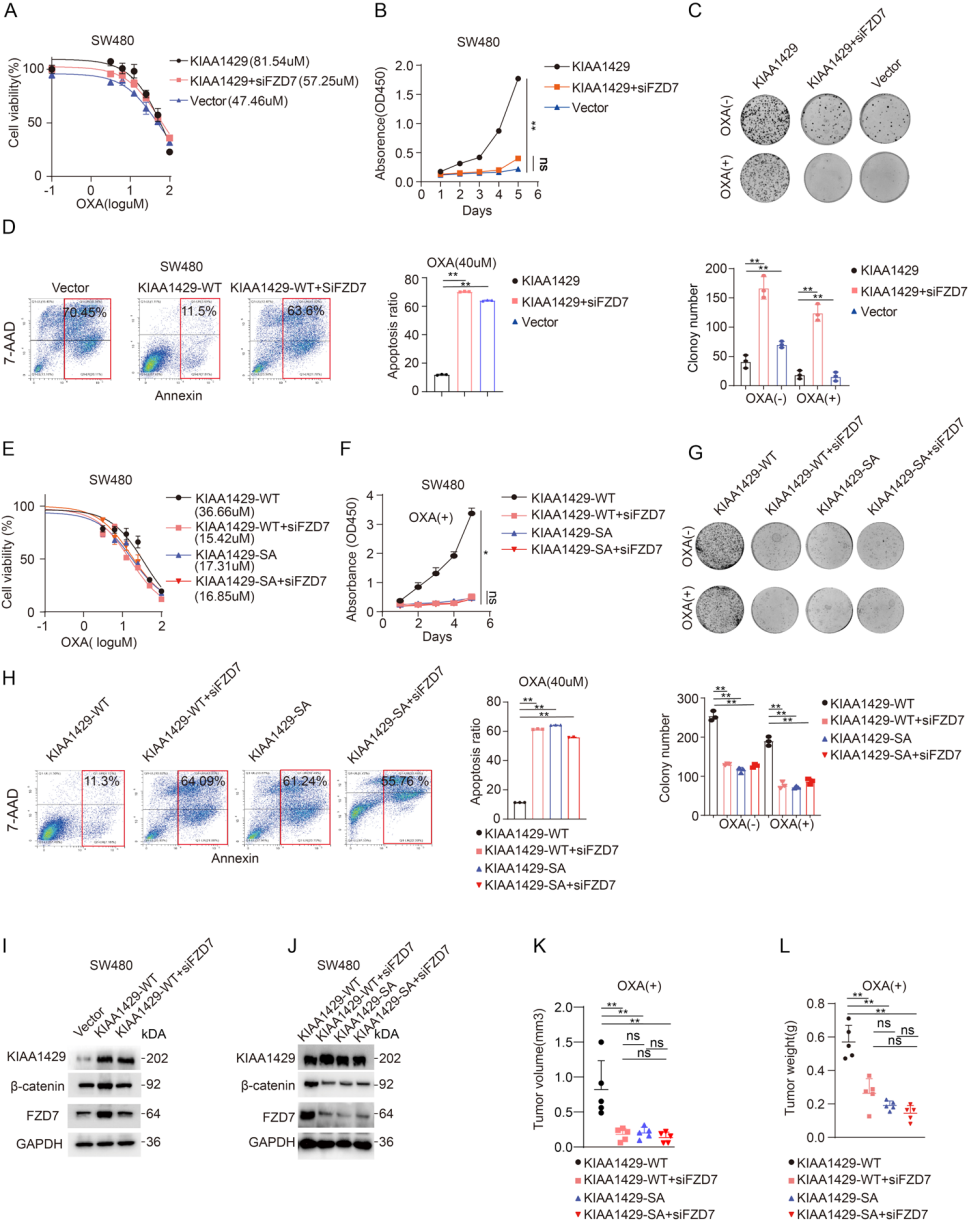
The effects of Cytoplasmic KIAA1429 on OXA resistance is dependent on FZD7 **(A)** The effect of siFZD7 in the KIAA1429 overexpressing SW480 cells as shown by IC50. **(B)** The effect of siFZD7 on the proliferation (1 μm OXA) of SW480 cells overexpressing KIAA1429 as shown by CCK8 assay. **(C)** The effect of siFZD7 on the proliferation (1 μm OXA) of SW480 cells overexpressing KIAA1429 as shown by colony formation assay. **(D)** The effect of siFZD7 on the proliferation (40 μm OXA) of SW480 cells overexpressing KIAA1429 as shown by flow cytometry analysis. **(E)** The effect of siFZD7 in the KIAA1429WT/KIAA1429SA overexpressing SW480 cells measured by IC50. **(F)** The effect of siFZD7 on the proliferation (1 μm OXA) of KIAA1429WT/KIAA1429SA overexpressing SW480 cells measured as shown by CCK8 assay. **(G)** The effect of siFZD7 on the proliferation (1 μm OXA) of KIAA1429WT/KIAA1429SA overexpressing SW480 cells measured as shown by colony formation assay. **(H)** The effect of siFZD7 on the proliferation (40 μm OXA) of KIAA1429WT/KIAA1429SA overexpressing SW480 cells measured as shown by flow cytometry analysis. **(I)** WB analysis showed the effect of siFZD7 on the expression of β-catenin of KIAA1429WT overexpressing SW480 cells. **(J)** WB analysis showed the effect of siFZD7 on the expression of β-catenin of KIAA1429WT/KIAA1429SA overexpressing SW480 cells. **K-L.** The effect of siFZD7 on the growth of KIAA1429WT/KIAA1429SA overexpressing SW480 cells, which were subcutaneously implanted in nude mice (*n* = 6), in the presence of OXA treatment (5 mg/kg OXA). Data were analyzed by one-way ANOVA adjusted for multiple comparisons for B, C,D, F,G, H,K, L

**Fig. 7:**
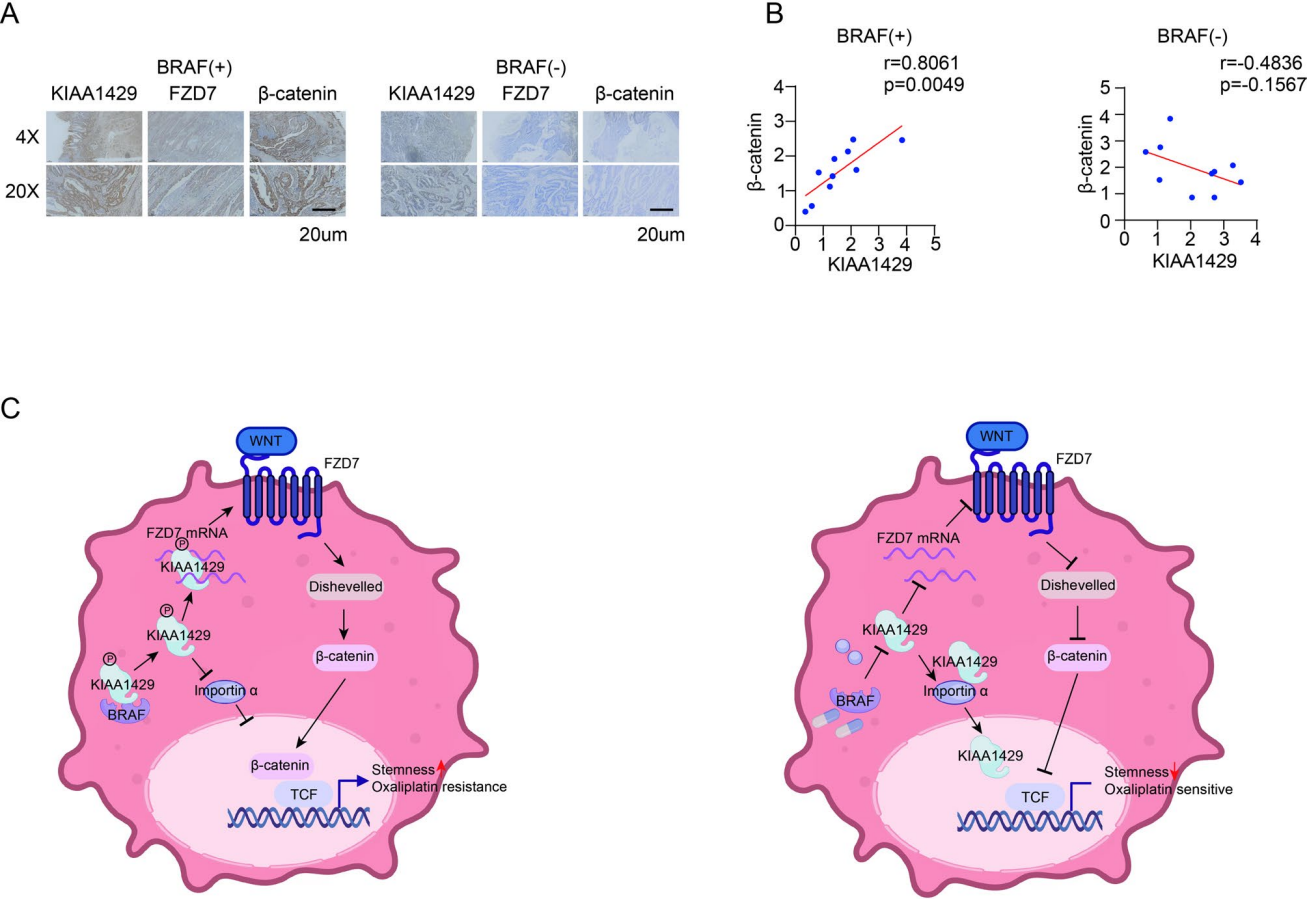
**(A)** Representative immunohistochemical images of BRAF-positive and BRAF-negative patients. **(B)** Correlation statistics of immunohistochemical scoring. **(C)** Working model

FZD7 has been confirmed as a downstream target gene of KIAA1429. However, the role of the KIAA1429-FZD7 axis in oxaliplatin resistance has not yet been verified. We found that in the SW480 cell line overexpressing wild-type KIAA1429, the IC50 of oxaliplatin significantly decreased after knocking down FZD7 with siRNA (Fig. 6A). Both CCK8 assay and colony formation also confirmed that knocking down FZD7 abolished the pro-proliferative effect of KIAA1429 (Fig. 6B-C). Knocking down FZD7 also rescued oxaliplatin-induced apoptosis, which was detected by FACS (Fig. 6D). The role of KIAA1429-FZD7 axis in oxaliplatin resistance also be confirmed by the colony formation, FACS and WB analysis in WiDr cell line (Fig. S6 A-C). In summary, these results confirm the role of the KIAA1429-FZD7 axis in oxaliplatin resistance in CRC. At the same time, phosphorylation modification on KIAA1429 has been confirmed to be a key factor affecting its function. The role of the phosphorylation modification site of KIAA1429 in oxaliplatin resistance was further verified. We overexpressed KIAA1429-WT and KIAA1429-SA plasmids respectively in the SW480 cell line, and then knocked down FZD7 respectively. The results showed that whether FZD7 was knocked down or not in the KIAA1429-SA group, the original function of KIAA1429 in promoting oxaliplatin resistance was lost, which was confirmed in both CCK8, FACS, colony formation and IC50 experiments (Fig. 6E-H). The same results were also be verified in WiDr cell line (Fig S6D-F). The WB analysis showed that knockdown of FZD7 counteracted the upregulation of β-catenin induced by KIAA1429-WT overexpression (Fig. 6I; S6E). Notably, β-ctaenin upregulation occurred exclusively in the presence of KIAA1429-WT (Fig. 6J; S6C). In the animal subcutaneous tumor experiment, the significant growth trend of tumor weight and volume only existed in the KIAA1429-WT group (Fig. 6K-L, Fig S6G, S7). We selected postoperative pathological sections from a group of patients who were BRAF-positive and BRAF-negative, respectively, and performed immunohistochemical staining. In the BRAF-positive patients, KIAA1429 was significantly positively correlated with β-catenin, while no significant correlation was observed in the BRAF-negative patients (Fig. 7A-B). Collectively, these results imply that the cytoplasmic KIAA1429 depends on FZD7 to promote oxaliplatin resistance, and maintaining its phosphorylation is key to its function.

## Discussion

Even though oxaliplatin-based chemotherapy is the conventional treatment for CRC, a significant number of patients will eventually develop resistance and experience recurrence. Therefore, pinpointing the crucial molecular driver events linked to chemoresistance is essential to strive for novel therapeutic strategies. These new strategies are vital for improve patients’ survival. In this study, we identified KIAA1429 is a key molecule for predicting the efficacy of neoadjuvant therapy and prognosis in CRC, and found that BRAF can regulate its phosphorylation modification and change its localization, thereby further upregulating the WNT pathway to promote oxaliplatin resistance. KIAA1429 as a core component in m^6^A writer complex, its oncogenic role in multiple cancer types has been investigated. For instance, KIAA1429 has been proven to promote the progression of Ewing sarcoma through STAT3 [15]. In liver cancer, though its m^6^A modification function, KIAA1429 promotes cell proliferation and metastasis by GATA3 [23]. Moreover, KIAA1429 was also been identified as a key driver for lung cancer gefitinib resistance [24]. Among these studies, what caught our attention the most is the study performed by Tariq M Rana. The study found that the different distribution of the KIAA1429 molecule in breast cancer is significantly correlated with staging and degree of malignancy [17]. Thus, we also observed this phenomenon in the neoadjuvant therapy for colorectal cancer and explored the underlying mechanisms. Importin α5 are key factors in altering its distribution and, in the presence of phosphorylation, continuously activate the WNT pathway in the cytoplasm. Targeting nuclear transport proteins to overcome therapy resistance may be a new direction for our further research.

FZD7, as a canonical receptor of the WNT pathway, has been proven to promote the development of pluripotent stem cells and to regulate therapy resistance in multiple cancer types [25–27].This study confirmed that KIAA1429 maintains the stability of FZD7 in a m^6^A-dependent manner to promote oxaliplatin resistance in CRC. The WNT pathway, characterized by the nuclear translocation of activated β-catenin, regulates downstream stemness genes. Early studies have also explored the significant activation of the WNT pathway through the action of FZD7 in BRAF inhibitor-resistant melanoma cells. This finding suggests that the interaction between BRAF and the WNT pathway may occur through FZD7 [28]. Additionally, studies have explored the development of inhibitors that specifically target FZD7 and future inhibitors targeting FZD7 may represent another therapeutic approach for colorectal cancer [29].

Phosphorylation is the most common type of post-translational modification of proteins, regulating numerous functions in tumor development and progression [30].This study, for the first time, has identified KIAA1429 as a novel substrate of BRAF, with BRAF regulating the function of KIAA1429 via phosphorylation. Kinases responsible for phosphorylation modifications are frequently investigated, and targeting these upstream regulators is widely considered a promising strategy in cancer treatment. Vemurafenib, a BRAF inhibitor, has been applied in various clinical trials for the treatment of CRC [31]. Therefore, this study may provide some guidance for therapy in patients with BRAF-mutant CRC.

In conclusion, this study has identified the role of the different nuclear and cytoplasmic distribution of KIAA1429 in oxaliplatin resistance in CRC. Mechanistically, BRAF phosphorylates KIAA1429 at serine 1579 and regulate its distribution. The accumulation of KIAA1429 in the cytoplasm through maintaining the RNA stability of FZD7 to continuously upregulate the WNT pathway. Our study provides guidance for the treatment and prognosis of CRC.

## Electronic supplementary material

Below is the link to the electronic supplementary material.

Supplementary Material 1

Supplementary Material 2

## Data Availability

No datasets were generated or analysed during the current study.

## Abbreviations

CRC: Colorectal cancer
KIAA1429/VIRMA: vir like m^6^A methyltransferase associated
OS: Overall survival
DFS: Disease-free survival
BARF: B-Raf proto-oncogene, serine/threonine kinase
nCRT: neoadjuvant therapy
TRG: Tumor regression grade
OXA: Oxaliplatin
pS/T: pan serine/threonine phosphorylation
FZD7: frizzled class receptor 7
m^6^A: N6-Methyladenosine
