# Supplementary material for: Phosphorylation of KIAA1429 promotes oxaliplatin resistance through activating the FZD7-Wnt signaling in BRAFV600E-mutated colorectal cancer
Source: J Exp Clin Cancer Res. 2025 Jul 3;44:187. doi: 10.1186/s13046-025-03449-w (PMC12225155; doi:10.1186/s13046-025-03449-w)
Supplement: Supplementary file 2 — Supplementary Material 2 [file 13046_2025_3449_MOESM2_ESM.docx]

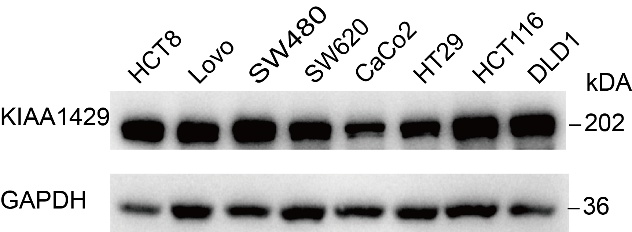


Figure S1. WB analysis of the expression of KIAA1429 in several CRC cell lines.


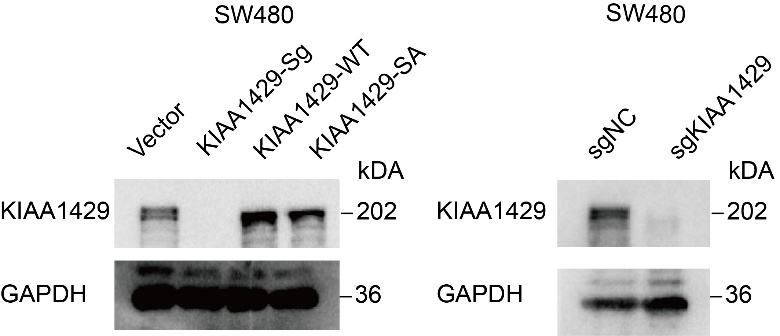


Figure S2. WB analysis to verify the Knockout and Overexpression of KIAA1429


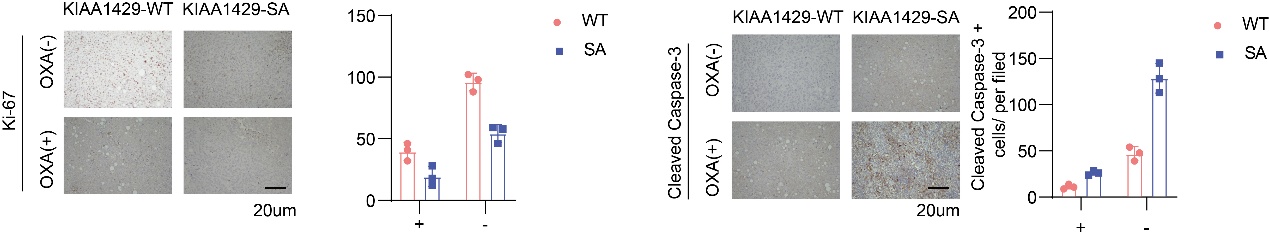


Figure S3. Immunohistochemistry of Ki-67 and cleaved caspase-3 from subcutaneous tumor.


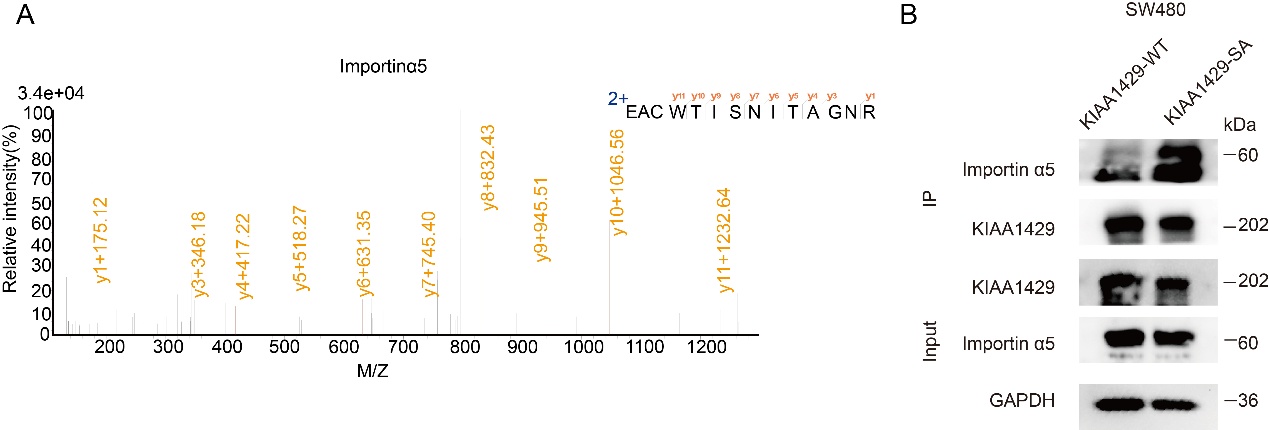


Figure S4. **A.** Fragmentation spectrum of the Importinα5 peptide identified by liquid chromatography/tandem mass spectrometry (LC-MS/MS) **B.** WB analysis showed that KIAA1429-SA has a stronger binding affinity with importin α5 compared to KIAA1429-WT (n = 3 biologically independent samples).


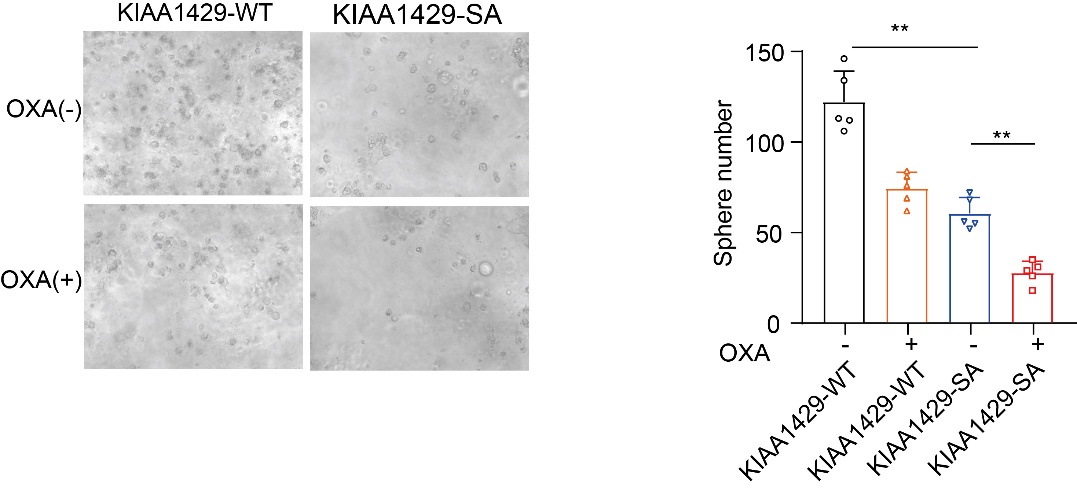


Figure S5. The Sphere formation assay of KIAA1429-WT/KIAA1429-SA Overexpression under in the presence/absence of Oxaliplatin (40 μMOXA). Data were analyzed by one-way ANOVA adjusted for multiple comparisons.


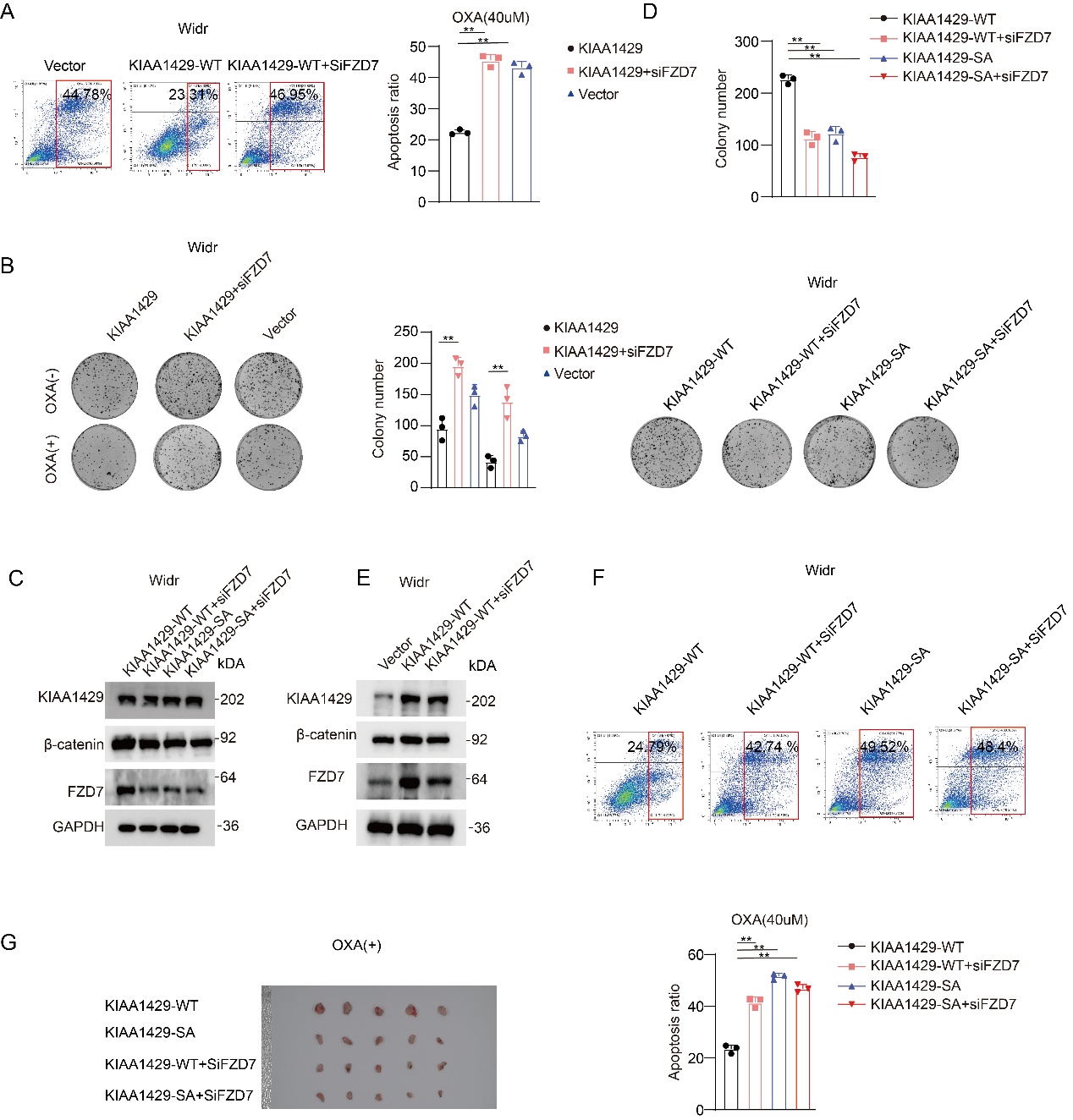


Figure S6. **A.** The effect of siFZD7 on the proliferation (40 μMOXA) of Widr cells overexpressing KIAA1429 as shown by flow cytometry analysis. **B.** The effect of siFZD7 on the proliferation (1 μMOXA) of Widr cells overexpressing KIAA1429 as shown by colony formation assay. **C.** WB analysis showed the effect of siFZD7 on the expression of β-catenin of KIAA1429-WT/KIAA1429-SA overexpressing Widr cells. **D.** The effect of siFZD7 on the proliferation (1 μMOXA) of KIAA1429-WT/KIAA1429-SA overexpressing WiDr cells measured as shown by colony formation assay. **E.** WB analysis showed the effect of siFZD7 on the expression of β-catenin of KIAA1429-WT/KIAA1429-SA overexpressing Widr cells. **F.** The effect of siFZD7 on the proliferation (40 μMOXA) of KIAA1429-WT/KIAA1429-SA overexpressing Widr cells measured as shown by flow cytometry analysis. **G.** Subcutaneous tumor to confirm the effect of siFZD7 on the growth of KIAA1429-WT/KIAA1429-SA overexpressing SW480 cells, which were subcutaneously implanted in nude mice (n = 6), in the presence of OXA treatment (5 mg/kg OXA). Data were analyzed by one-way ANOVA adjusted for multiple comparisons A,B,D,F.


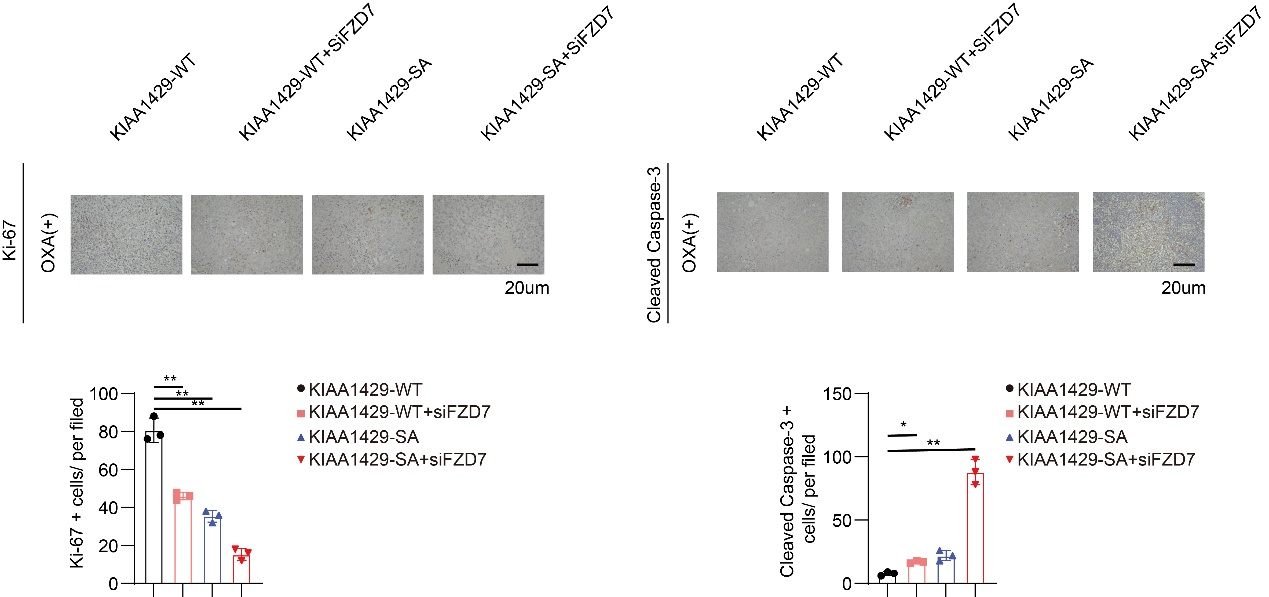


Figure S7. Immunohisochemistry of Ki-67 and cleaved caspase-3 from subcutaneous tumor. Data were analyzed by one-way ANOVA adjusted for multiple comparisons
